# Role of Lung Ultrasound in the Detection of Lung Sequelae in Post-COVID-19 Patients: A Systematic Review and Meta-Analysis

**DOI:** 10.3390/jcm13185607

**Published:** 2024-09-21

**Authors:** Andrea Boccatonda, Damiano D’Ardes, Viola Tallarico, Maria Teresa Guagnano, Francesco Cipollone, Cosima Schiavone, Fabio Piscaglia, Carla Serra

**Affiliations:** 1 Department of Medical and Surgical Sciences, University of Bologna, 40126 Bologna, Italy; fabio.piscaglia@unibo.it; 2 Diagnostic and Therapeutic Interventional Ultrasound Unit, IRCCS Azienda Ospedaliero-Universitaria di Bologna, 40138 Bologna, Italy; carla.serra@aosp.bo.it; 3 Department of Medicine and Aging Science, Institute of “Clinica Medica”, “G. d’Annunzio” University of Chieti, 66100 Chieti, Italy; damiano.dardes@unich.it (D.D.); guagnano@unich.it (M.T.G.); francesco.cipollone@unich.it (F.C.); 4 Internal Medicine, Bentivoglio Hospital, Azienda Unità Sanitaria Locale (AUSL) Bologna, 40010 Bentivoglio, Italy; viola.tallarico@ausl.bo.it; 5 Internistic Ultrasound Unit, SS Annunziata Hospital, “G. d’Annunzio” University of Chieti, 66100 Chieti, Italy; cosima.schiavone@gmail.com; 6 Division of Internal Medicine, Hepatobiliary and Immunoallergic Diseases, IRCCS Azienda Ospedaliero-Universitaria di Bologna, 40138 Bologna, Italy

**Keywords:** lung, ultrasound, post-COVID, long-COVID, chest

## Abstract

**Background**: During the COVID-19 pandemic, several studies demonstrated the effectiveness of lung ultrasound (LUS) as a frontline tool in diagnosing and managing acute SARS-CoV-2 pneumonia. However, its role in detecting post-COVID-19 lung sequelae remains to be fully determined. This study aims to evaluate the diagnostic accuracy of LUS in identifying lung parenchymal damage, particularly fibrotic-like changes, following COVID-19 pneumonia, comparing its performance to that of CT. **Methods**: Relevant studies published before July 2024 were identified through a comprehensive search of PubMed, Embase, and Cochrane library. The search terms were combinations of the relevant medical subject heading (MeSH) terms, key words and word variants for “lung”, “post-COVID”, “long-COVID”, and “ultrasound”. The pooled sensitivity, specificity, diagnostic odds ratio (DOR), and summary receiver-operating characteristic (SROC) curve were used to examine the accuracy of CEUS. The selected works used different thresholds for the detection and counting of B-lines by ultrasound. This led to dividing our analysis into two models, the first based on the lower thresholds for detection of B-lines found in the works, and the second on data obtained using a higher detection threshold. **Results**: In terms of the diagnostic accuracy of LUS in detecting residual fibrotic-like changes in patients post-COVID-19 infection, a low-threshold model displayed a pooled sensitivity of 0.98 [95% confidence interval (CI): 0.95–0.99] and a pooled specificity of 0.54 (95% CI: 0.49–0.59). The DOR was 44.9 (95% CI: 10.8–187.1). The area under the curve (AUC) of SROC was 0.90. In the second analysis, the model with the higher threshold to detect B-lines showed a pooled sensitivity of 0.90 (95% CI: 0.85–0.94) and a pooled specificity of 0.88 (95% CI: 0.84–0.91). The DOR was 50.4 (95% CI: 15.9–159.3). The AUC of SROC was 0.93. **Conclusions**: In both analyses (even using the high threshold for the detection of B-lines), excellent sensitivity (98% in model 1 and 90% in model 2) is maintained. The specificity has a significant variation between the two models from 54 (model 1) to 87% (model 2). The model with the highest threshold for the detection of B-lines displayed the best diagnostic accuracy, as confirmed by the AUC values of the SROC (0.93).

## 1. Introduction

In previous viral outbreaks like those caused by MERS-CoV and SARS-CoV, a varying proportion of survivors developed interstitial lung disease (ILD), including pulmonary fibrosis (PF) [1,2,3]. Recent studies highlighted early respiratory complications following COVID-19, including persistent symptoms, impaired lung function, and interstitial lung abnormalities [4,5,6,7]. Histological examinations of lung biopsies from these patients suggest the presence of organizing pneumonia and pneumonitis [8]. However, the extent, severity, and potential reversibility of these post-COVID-19 conditions are still unclear, emphasizing the need for ongoing monitoring after COVID-19 pneumonia. While several follow-up protocols have been proposed, they vary widely in their recommended diagnostic procedures [9,10,11,12]. For instance, although chest imaging is universally recommended during the initial assessment, there is no clear consensus on whether chest radiography (CXR) or computed tomography (CT) should be used. Notably, lung ultrasound (LUS) has been considered in some studies [13,14,15]. In recent decades, LUS has emerged as a viable alternative to traditional radiological methods for various lung diseases [16,17,18,19,20,21,22,23,24]. During the COVID-19 pandemic, numerous studies have demonstrated the effectiveness of LUS as a frontline tool in diagnosing and managing acute SARS-CoV-2 pneumonia [16,25]. However, its role in detecting post-COVID-19 interstitial sequelae remains to be fully determined [26,27,28].

A recent meta-analysis by Guinto et al. performed on 47 studies and 3557 patients demonstrated that the most prevalent computed tomography (CT) imaging abnormality was ground-glass opacities (GGOs) (44.9% (95% CI 37.0–52.9%) at any follow-up time point [29]. The occurrence of reticulations significantly decreased between early and late follow-ups [29]. 

LUS has already been validated for detecting ILD secondary to other causes, with several studies, particularly those involving ILD associated with connective tissue diseases, showing that LUS is superior to CXR and, importantly, offers similar sensitivity and negative predictive value compared to CT [17,30,31]. Therefore, ultrasound is a promising first-line diagnostic tool due to its non-invasive, non-ionizing, and cost-effective nature in the evaluation of patients after COVID-19 acute infection. 

This study aims to evaluate the diagnostic accuracy of LUS in identifying lung parenchymal damage, particularly fibrotic-like changes, following COVID-19 pneumonia, comparing its performance to that of CT. The systematic review was registered with PROSPERO CRD42024586785; we registered the work as “started review”. We followed PRISMA reporting guidelines [32].

## 2. Material and Methods

### 2.1. Eligibility Criteria 

Inclusion and exclusion criteria were set before the literature search. Studies were selected if satisfied these criteria: clinical studies focused on the diagnostic value of ultrasound for the diagnosis of lung parenchymal damage following COVID-19 pneumonia; the gold reference standard for diagnosis was chest CT; data were sufficient enough to construct a 2 × 2 contingency table for true positives (TP), false positives (FP), true negatives (TN), and false negatives (FN); informed consents were obtained from each patient and approved by ethics committee; articles written in English. Studies were excluded if met these criteria: reviews, editorial articles, or case reports; studies lacking necessary data.

### 2.2. Information Sources

Relevant studies published before July 2024 were identified through a comprehensive search; three electronic databases were searched including MEDLINE (including Cochrane Library) and EMBASE.

### 2.3. Search Strategy

The search terms were combinations of the relevant medical subject heading (MeSH) terms, keywords and word variants for “lung”, “post-COVID”, “long-COVID”, and “ultrasound”. We used the following search strategy, adapted for the requirements of each database if necessary: (“post-COVID-19” OR “post-2019-nCov” OR “post-SARS-CoV-2”) AND (“lung ultrasonography” OR “lung ultrasound” OR “lung echography” OR “lung ultrasonics” OR “lung ultrasonic diagnosis” OR “lung ultrasonic echo” OR “lung ultrasonic examination” OR “lung ultrasonic scanning”). The details of the search strategy are shown in Appendix A.

### 2.4. Selection Process

The title and abstract of each study were reviewed first, then the full text was read to further screen the articles. In addition, the references of each retrieved article were manually screened to identify other potentially eligible studies. 

### 2.5. Data Collection Process

Data extraction was conducted by 2 researchers independently (A.B. and V.T.). Divergences were assessed by a third reviewer (D.D.).

### 2.6. Data Items

Data extraction was conducted including the first author’s name, the publication year of the study, study design, number of patients, mean age of patients, follow-up timing, male patients, healthy patients, pathological patients, standard reference, LUS scheme, LUS interpretation and type of probe. 

### 2.7. Study Risk of Bias Assessment

To assess the methodological quality of the included studies, Quality Assessment of Diagnostic Accuracy Studies (QUADAS) tool was used by 2 researchers independently, the form of which was constituted of 14 questions [33]. For each item, the study was rated as “yes” (high quality) if reported; “no” (low quality) if not reported; “unclear” if no adequate information was provided. Disagreements were also resolved by a third researcher. Detailed information regarding sample size, age, gender, and reference standards in individual studies are shown in Table 1.

### 2.8. Effect Measures

In each selected study, true positive (TP), true negative (TN), false positive (FP), and false negative (FN) were collected directly or calculated according to the sensitivity, specificity, positive predictive value (PPV), and negative predictive value (NPV). 

### 2.9. Synthesis Methods, Reporting Bias Assessment and Certainty Assessment

All the statistical analyses were performed by RevMan 5.0 and Meta-Disc Version 1.4 (Unit of Clinical Biostatistics team of the Ramony Cajal Hospital, Madrid, Spain). A summary of sensitivity, specificity, positive likelihood ratios (PLR), negative likelihood ratios (NLR), and diagnostic odds ratio (DOR) were calculated from the TP, FP, FN, and TN of each study, which indicated the accuracy of ultrasound in detecting lung parenchymal damage. Meanwhile, the summary receive-operating characteristics (SROC) curve was constructed as described by Moses et al. to summarize the TP and FP rates [34]. The inconsistency index (I^2^) was used to detect the heterogeneity among different studies [35]. For heterogeneity categorization, we defined an I^2^ of less than 25% as low, 25% to 49% as low to moderate, 50% to 74% as moderate to high, and 75% or above as high. When I^2^ > 50% revealed considerable heterogeneity (16), we would proceed with our analysis using a random effects model. Publication bias was assessed by a contour-enhanced funnel plot, and *p* > 0.05 was considered no significant publication bias [36].

## 3. Results

### 3.1. Study Identification

**Figure 1:**
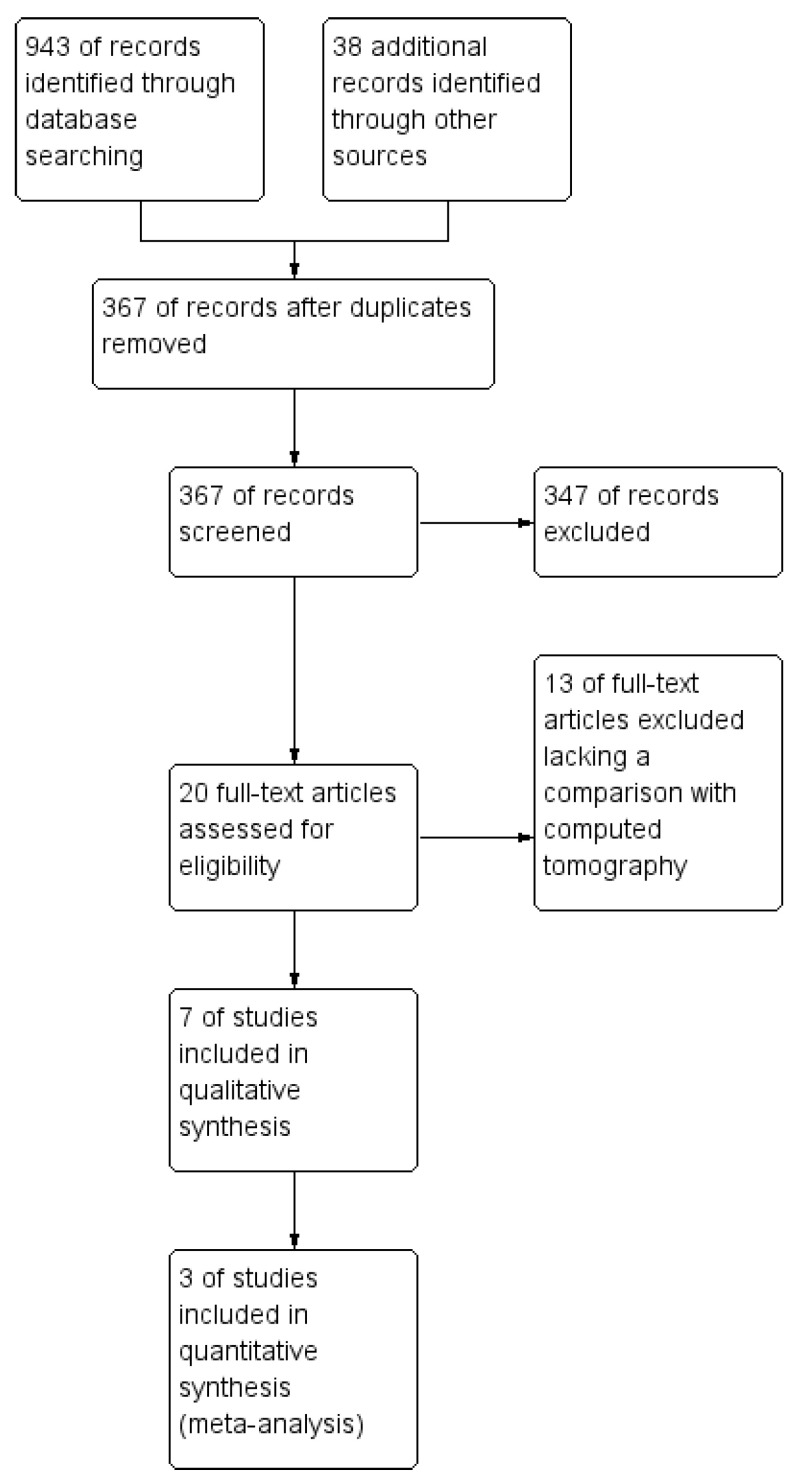
Study flow diagram.

After duplicates removal, a total of 367 potentially relevant articles were identified in the initial search stage. Most of these papers were excluded due to titles and abstracts. Only 20 studies were chosen for full-text review, and 13 studies were excluded lacking the comparison with the reference standard method (chest computed tomography) [13,14,15,37,38,39,40]. Further identification excluded four articles lacking data to construct a 2 × 2 contingency table. Finally, three articles satisfying the inclusion criteria were included and analyzed [13,14,15]. The demonstration of this study search flow can be seen in Figure 1.

### 3.2. Study Characteristics

The study characteristics of all included studies are demonstrated in Table 1. In total, 610 patients at 3 months post-COVID-19 acute infection were analyzed. Particularly, 411 patients (67.3%) had no pulmonary sequelae on follow-up chest CT. The remaining 199 patients (32.6%) had persistent pulmonary changes on a 3-month chest CT follow-up.

### 3.3. Quality Assessment

**Figure 2:**
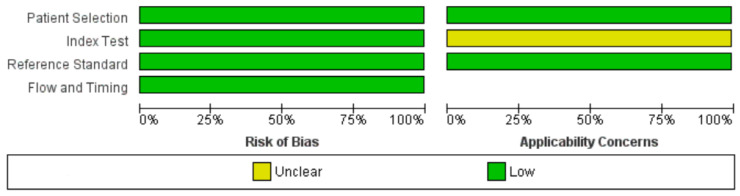
Risk of bias and applicability concerns graph: review authors’ judgments about each domain presented as percentages across included studies.

**Figure 3:**
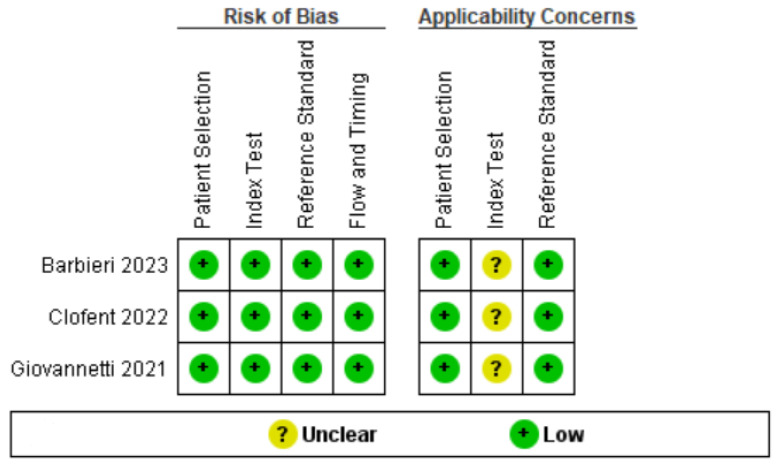
Risk of bias and applicability concerns summary: review authors’ judgments about each domain for each included study [13,14,15].

The three selected studies were characterized by a low risk of bias in almost all evaluated items [13,14,15] (Figure 2 and Figure 3). Both authors who reviewed and evaluated the works, and a third expert author, judged to place an “uncertain” in the category of applicability of the index test. This is because the three selected works used different thresholds for the detection and counting of B-lines by ultrasound [13,14,15]. This led to dividing our analysis into two models, the first based on the lower thresholds for detection of B-lines found in the works, and the second on data obtained using a higher detection threshold. Detailed information regarding sample size, age, gender, and reference standards in individual studies are shown in Table 1.

### 3.4. Overall Diagnostic Accuracy of Lung Ultrasound to Detect Fibrotic-like Changes (MODEL 1)

**Figure 4:**
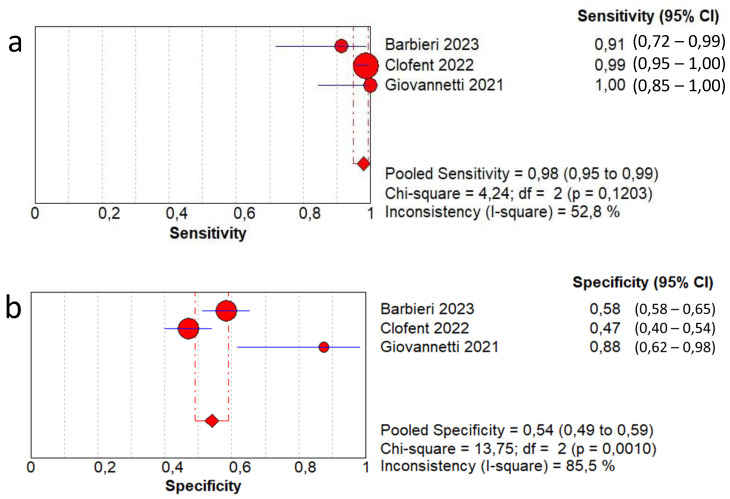

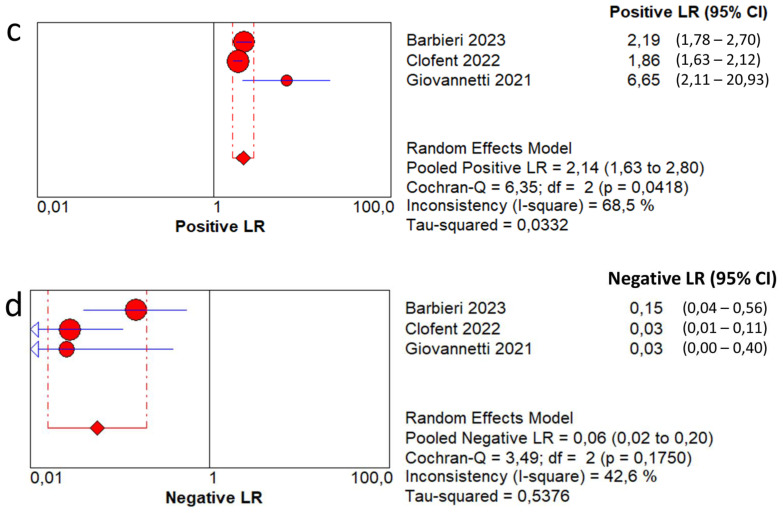

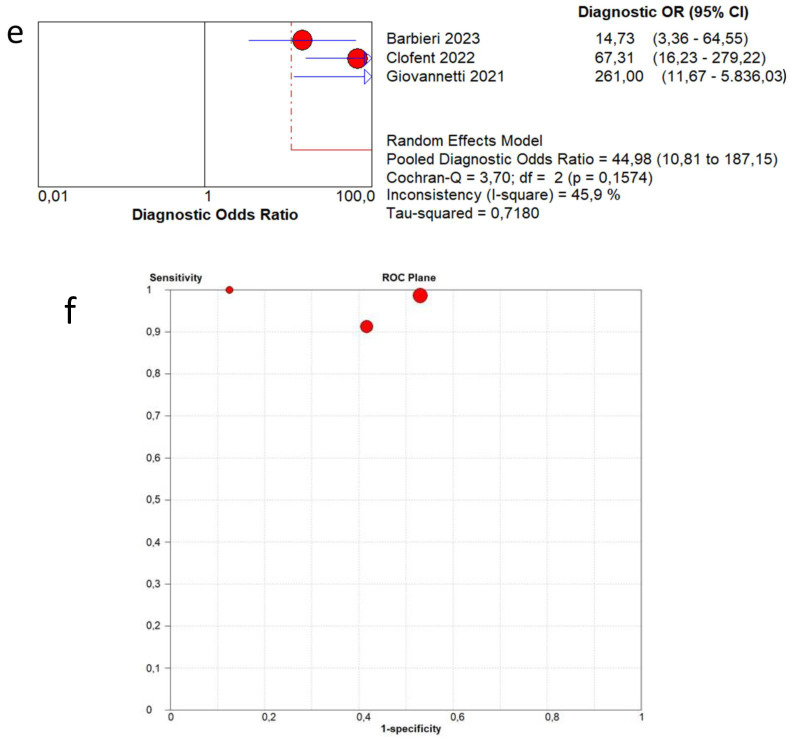

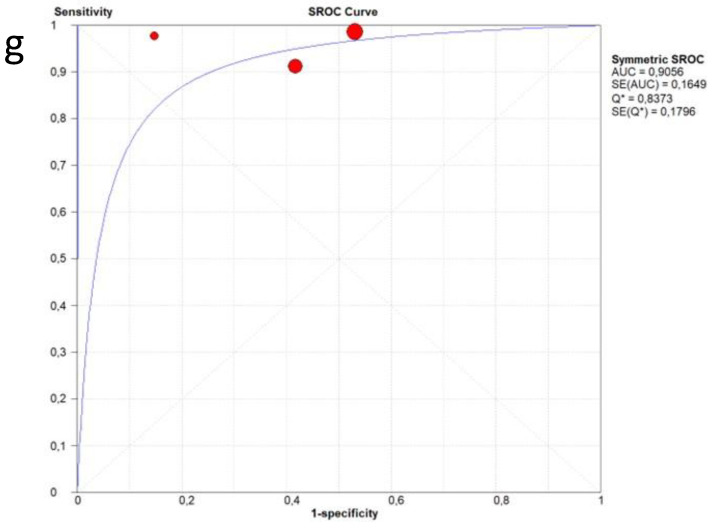
First analysis by using a low-threshold lung ultrasound model to detect fibrotic-like changes in post-COVID-19 patients in comparison with chest computed tomography. (**a**) Sensitivity; (**b**) specificity; (**c**) positive likelihood-ratio (LR); (**d**) negative LR; (**e**) diagnostic odds ratio; (**f**) ROC curve; (**g**) SROC curve [13,14,15].

In terms of the diagnostic accuracy of lung ultrasound in detecting residual fibrotic-like changes in patients post-COVID-19 infection, the pooled sensitivity was 0.98 (95% CI: 0.95–0.99) and the pooled specificity was 0.54 (95% CI: 0.49–0.59). The DOR was 44.9 (95% CI: 10.8–187.1) with a low to moderate heterogeneity (I^2^ = 45.9%). The AUC of SROC was 0.90. A moderate heterogeneity was found in sensitivity (I^2^  =  52.8%) and a high heterogeneity in specificity (I^2^  =  85.5%) (Figure 4).

### 3.5. Overall Diagnostic Accuracy of Lung Ultrasound to Detect Fibrotic-like Changes (MODEL 2)

**Figure 5:**
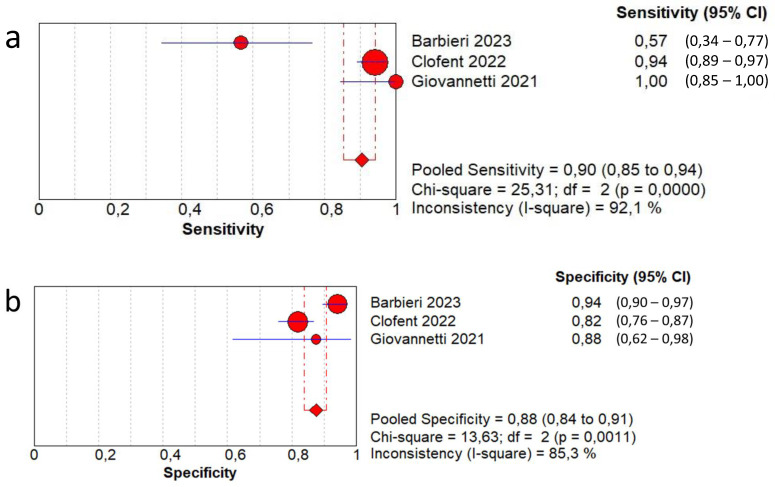

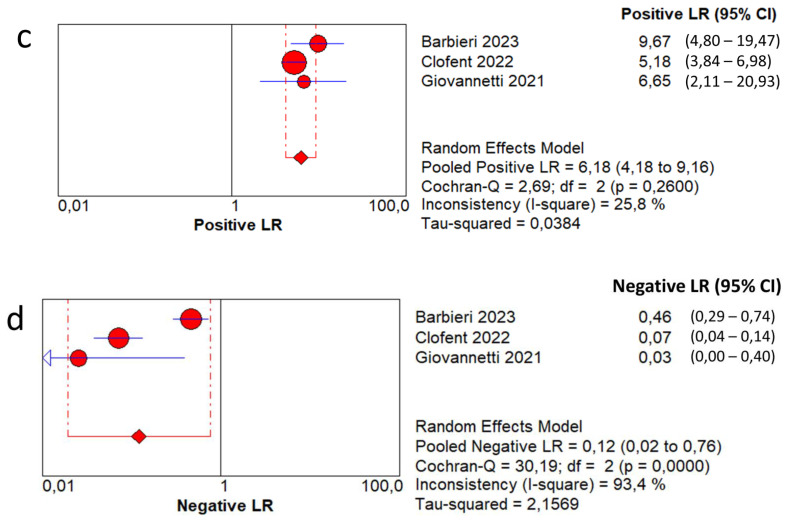

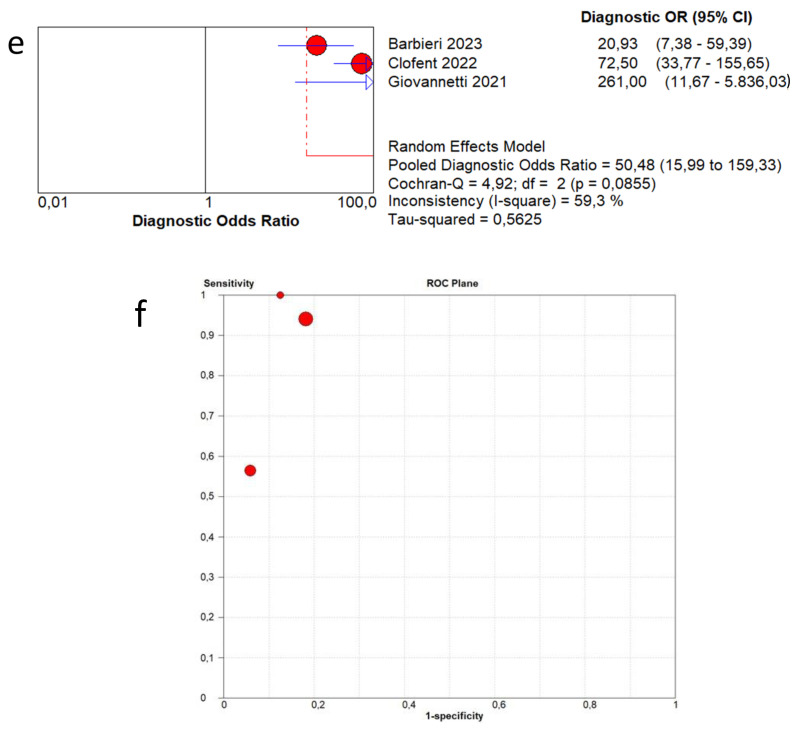

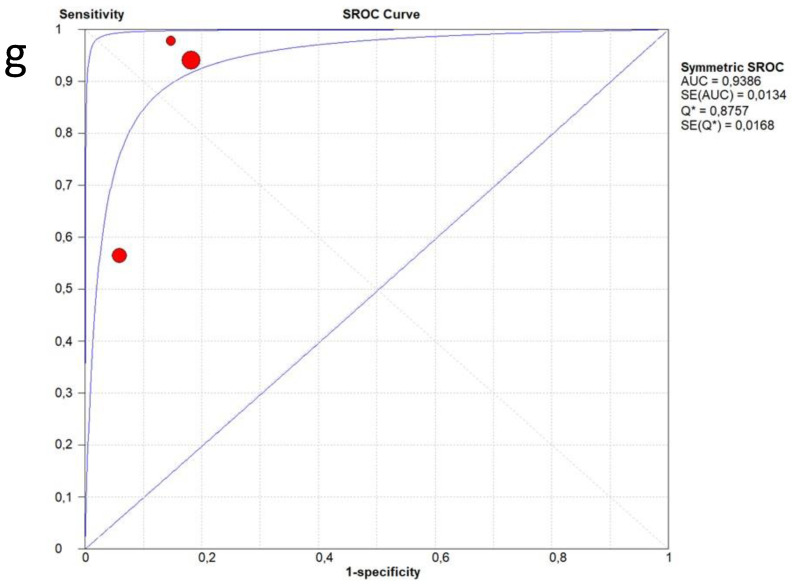
First analysis by using a high-threshold lung ultrasound model to detect fibrotic-like changes in post-COVID-19 patients in comparison with chest computed tomography. (**a**) Sensitivity; (**b**) specificity; (**c**) positive likelihood-ratio (LR); (**d**) negative LR; (**e**) diagnostic odds ratio; (**f**) ROC curve; (**g**) SROC curve [13,14,15].

In terms of the diagnostic accuracy of lung ultrasound in detecting fibrotic-like changes in patients after COVID-19 infection in comparison with CT scan, the pooled sensitivity was 0.90 (95% CI: 0.85–0.94) and the pooled specificity was 0.88 (95% CI: 0.84–0.91). The DOR was 50.4 (95% CI: 15.9–159.3) with a moderate heterogeneity (59.3%). The AUC of SROC was 0.93. High heterogeneity was found in sensitivity (I^2^  =  92.1%) and in specificity (I^2^  =  85.3%) (Figure 5).

### 3.6. Publication Bias

**Figure 6:**
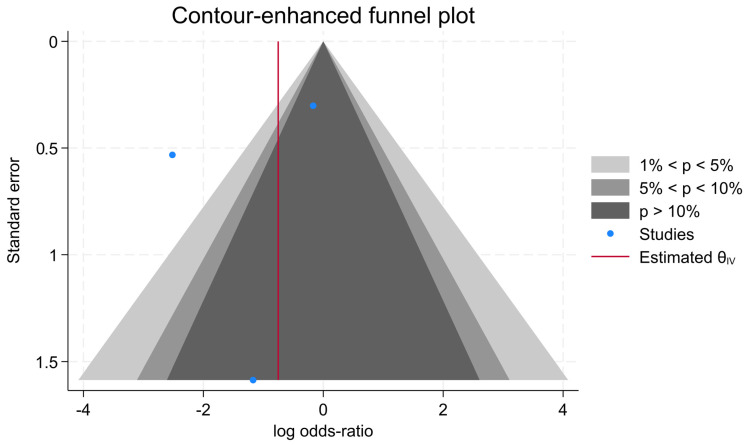
Contour-enhanced funnel plot of the three evaluated studies.

The results of the contour-enhanced funnel plot confirmed that no publication bias was observed among the studies in CEUS (*p*  <  0.01) (Figure 6).

## 4. Discussion

LUS found wide use in the most complex and difficult phases of the various waves of the SARS-CoV-2 virus pandemic due to its simplicity of execution, reproducibility, speed of execution, low cost, and no exposure to radiation [25]. A recent Cochrane systematic review and meta-analysis highlighted how the diagnostic accuracy of LUS is superior to that of a chest X-ray and almost comparable to that of a chest CT in identifying the various signs and pathological findings of COVID-19 pneumonia [41]. Particularly, chest CT and ultrasound display higher sensitivity estimates than X-ray (*p* = 0.0003 and *p* = 0.001, respectively) [41]. Chest CT and ultrasound gave similar sensitivities (*p* = 0.42). All modalities had similar specificities (CT versus X-ray *p* = 0.36; CT versus ultrasound *p* = 0.32; X-ray versus ultrasound *p* = 0.89) [41].

COVID-19 pneumonia presents particular but not pathognomonic ultrasound signs [25]; therefore, ultrasound does not allow a specific etiological diagnosis of acute SARS-CoV-2 infection but only to evaluate the possible lung involvement; indeed, the diagnosis should be performed through a nasopharyngeal swab still almost 5 years after the beginning of the spread of the virus. The main ultrasound finding of an acute COVID-19 infection (pneumonia) is that of a bilateral diffuse interstitial disease (B lines or vertical artifacts) with non-homogeneous distribution (patchy appearance) with irregularity of the pleural line, primarily involving the basal dorsal areas of the lungs; consolidative features appear in the most severely compromised lung areas [25]. In the first pandemic waves, where there was a very high spread and contagiousness of the virus, to finding a picture of interstitial disease in a patient with fever and other compatible symptoms was highly suggestive of acute COVID-19 infection. With the progression of the pandemic and the advent of new variants of the virus and vaccinations, there has been a change in the clinical presentation, with often pauci-symptomatic cases.

From an ultrasound and clinical point of view, the spread of COVID-19 has reinforced the concept that interstitial lung disease detected on ultrasound can have multiple pathological mechanisms and must be included in the differential diagnosis between cardiogenic, non-cardiogenic (ARDS), chronic interstitial lung disease and therefore infectious pneumonia (mainly viral ones) [25]. Furthermore, we have gradually moved to evaluate the condition of patients who healed from COVID-19 acute infection. In general, it is possible to affirm that although many of the patients recover without sequelae, in a series of subjects, interstitial-alveolar alterations similar to chronic pulmonary fibrosis may persist [42]. Imaging with severe post-COVID pulmonary fibrosis was found mostly after the first waves of the pandemic and in general in patients who had presented more serious clinical pictures during the acute phase (need for hospitalization in intensive care, intubation, high oxygen flows).

LUS scores correlated with the length of hospitalization, age, use of non-invasive ventilation, administration of corticosteroids therapy, and laboratory parameters during the acute phase such as white blood cell (WBC) count, platelet count, C-reactive protein (CRP), D-dimer, interleukin (IL)-6, and inversely correlated with lymphocyte count [16,43,44]. 

Several diagnostic imaging studies evaluated the pulmonary sequelae of COVID-19 infection, with serial timing from 1 to 3 months up to over 1 year [2,10,11,39,43,45]. Most of these studies were performed by chest CT [2,10,11,43]. In the literature, there are also several studies on lung ultrasound in the follow-up of post-COVID-19 patients [13,14,15,26,28,37,38,39,40,43,45,46,47,48,49,50,51,52]. Most of these are cohort studies based on ultrasound alone, with a series of correlations with clinical data [13,14,15,28,37,38,39,40,43,45,46,47,48,49,50,51]. Only seven of those studies have set up a comparison between the index method (ultrasound) and the reference standard (CT) [13,14,15,37,38,39,40]. As highlighted in our systematic review, three of these seven studies propose diagnoses and binominal data on the resolution or otherwise of post-COVID pulmonary involvement [13,14,15]. The other studies propose data with continuous values, with particular reference to the LUS score [37,38,39,40].

The term LUS score refers to a reporting scheme, based on a specific number of scan areas per hemithorax; for each lung area a score is assigned related to the evaluation of the damage (de-aeration) of the lung parenchyma, to determine a final numerical score [16,53,54]. In the literature, many works correlated this score (LUS score) to several clinical, laboratory, blood gas analysis and clinical outcomes parameters, showing how the same score is a risk/predictive factor for mortality and/or for severe respiratory failure [16,53,54]. The main limit in the diffusion and use of the LUS score is in its definition; indeed, several score models have been developed based on a different number of scan areas and on a different interpretation of the ultrasound signs; therefore, the different models were poorly comparable between different studies and research groups (see Table 2). The LUS score plays a relevant diagnostic role when repeated with the same methods over time [38,40,43].

In the works in which a serial analysis was performed over time, it was seen that the LUS score progressively decreases in cases of recovery and can maintain increased values in cases in which an interstitial disease persists, according to the degree of pulmonary changes.

In the work of Ramos Hernandez and colleagues, there was a significant improvement (reduction) in the lung score compared to the first visit (3 months to 12 months) (5.8 ± SD 5.2 vs. 2.1 ± SD 3.8; *p* = 0.001) [38].

In a work by Russo et al., a decrease in total score greater than 50% was observed in 76% of patients after 6 months when compared to LUS during hospitalization. LUS score < 2 can rule out fibrotic-like changes with a sensitivity of 0.92 (95% CI 0.73–0.99) and a specificity of 0.60 (95% CI 0.45–0.74); ROC analysis showed an AUC of 0.85 (95% CI 0.76–0.93) [39].

Other studies confirmed a decrease in LUS score values during follow-up, with good concordance with chest CT data [37,40].

The clinical question of our systematic review and meta-analysis was to evaluate whether lung ultrasound could exert a relevant role in the follow-up of patients after acute COVID-19 infection; in particular, to compare its diagnostic accuracy on the evaluation of residual lung damage (sequelae), in this category of patients, in comparison with the gold-standard method (CT scan).

From a methodological point of view, we performed two different analyses because in two of the three works analyzed, the authors used different cut-offs to quantify the degree of interstitial lung disease on ultrasound. The cut-off was linked to the evaluation of lung damage, therefore on the number of B lines or LUS score value. It was possible to evaluate a series of data based on less restrictive criteria (low threshold: LUS 3 points in Barbieri’s work [13] and any B line in Clofent’s work [14]) and a second analysis with a higher criterion (high threshold: LUS 7 points in Barbieri’s work [13] and >3 B lines per area in Clofent’s work [14]). In the first model, sensitivity values of 98% and specificity of 54% were obtained, with AUC SROC of 0.90. In the second analysis, sensitivity values of 90% and specificity of 87% were obtained, with AUC SROC of 0.93.

The most significant result is that in both analyses (even using the highest cut-offs for the number of B-lines), excellent sensitivity > 90% is maintained. As expected, the specificity has a significant variation between the two models from 54 to 87%. Therefore, the model with the highest cut-off for the definition of B-lines has the best diagnostic accuracy, as confirmed by the AUC values of the SROC (0.93).

### Other Pathological Lung Ultrasound Findings

The work of Clofent et al. highlighted how the fragmented pleural line was present in 7.8% of patients with residual lung damage and in no case of patients who had undergone complete recovery [14]; a thickened pleural line was present in 35.9% of patients without residual lung damage and in 77.3% of those with evident damage on chest CT [14]. In the work of Russo et al. 52.8% of patients presented thickened and fragmented pleural line at 6 months [39]. In Zimna’s work, the finding of an “irregular pleural line” increased from 56.9% to 38.3% from the first to the second follow-up, while the finding of a “broken pleural line” was present in 3.0% of the first follow-up to 2.3% of the second one [40].

Consolidations were found in 1.5% of the cases of patients without sequelae vs. 6.5% of patients with persistent pulmonary alterations at 3 months of follow-up in the work of Clofent et al. [14]. In the work of Russo et al. small subpleural nodules were present in 43% of the patients followed up at 6 months [39]. In the work of Zimna et al., the authors divided the consolidations into three categories according to size (limits 2.5 mm and 10 mm); consolidations between 2.5 and 10 mm were prevalent on both ultrasound checks (26.5% of the pulmonary areas examined in the first period and 13.8% of the second period) [40]. In the work of Giovannetti et al., neither pleural effusion nor consolidation were found in the patients followed up [15].

In Clofent’s work, there was no evidence of pleural effusion in subjects with recovery from pneumonia vs. 1.9% in cases with persistent lung involvement [14]. Zimna et al. showed pleural effusion to be present in 1% of the lung areas examined at both follow-up times [40]. Therefore, pleural effusion is a rare finding in post-COVID patients; it is important to underline that pleural effusion was also low frequent in acute COVID-19 pneumonia conditions.

## 5. Study Limitations

The main limitation of our study is the limited number of works consistent with our inclusion criteria. The fact of having analyzed only three works leads to inconsistency values often > 50%. Another main limitation of this study is the lack of standardized LUS protocols among different works; therefore, authors display different results according to many different thresholds for detecting B-lines. Moreover, LUS seems to be highly dependent on the sonographer’s experience.

In our meta-analysis, we tried to overcome this limitation by performing two analyses creating two B-line detection thresholds (high vs. low). 

Many of the published studies lack comparison with the reference method, which is currently chest CT. The majority of published studies performed 3–6 months follow-up. Future studies are necessary to extend this follow-up (12–24 months) to verify the long-term change in some pulmonary alterations, particularly in patients who continue to present symptoms (long-COVID).

## 6. Conclusions

In both analyses (even using the highest cut-offs for the number of B-lines), excellent sensitivity (over 90%) is maintained. The specificity has a significant variation between the two models from 54 (model 1) to 87% (model 2). The model with the highest threshold for the detection of B-lines displayed the best diagnostic accuracy, as confirmed by the AUC values of the SROC (0.93). Therefore, it is possible to conclude that LUS presents good diagnostic accuracy in the diagnosis of pulmonary organ damage (sequelae) in post-COVID-19 patients. LUS has the potential to enable standardized follow-up without radiation exposure and with lower associated costs in comparison to CT scans. Uncertainties remain on a common and standardized reporting method. Future studies and consensus will have to establish a shared strategy for ultrasound evaluation in this category of patients.

## Figures and Tables

**Table 1:** Summary of findings of the studies included in the meta-analysis.

Author	Year	Study Design	Patients	Age	Follow-Up Timing	Male	Healthy Patients	Pathological Patients	Standard Reference	LUS Scheme	LUS Interpretation	Probe
Barbieri et al. [13]	2023	Prospective	220	62.2	3 months	62.1%	197	23	CT	16 areas	Score 0 only A-lines or less than 3 separated B-lines; score 1 in case of 3 or more B-lines or coalescent B-lines occupying ≤ 50% of the screen; score 2 for coalescent B-lines occupying > 50% of the screen; and score 3 for consolidation. A final LUS score, achieved from the sum of all values obtained within the 16 areas can range from 0 to 48.	Convex probes (frequency 2.5–5 MHz)
Clofent et al. [14]	2022	Prospective	352	56.0	2−5 months	57.7%	198	154	CT	12 areas	B-line score by summing 1 point for each thoracic area with pathological B lines (score range, 0 to 12).	2–5 MHz convex transducer
Giovannetti et al. [15]	2021	Prospective	38	60.6	3 months	71.1%	16	22	CT	12 areas	Score 0 as normal pattern, A-lines or <3 B-lines; 1 as moderate loss, ≥3 B-lines; 2 as severe loss, coalescent B-lines; 3 as complete loss, white lung and/or lung consolidations. The total LUS score was the sum of the points from each lobe and ranges from 0 to 36 points.	Linear array probe (MHz 7.5–10)

**Table 2:** Summary of findings of other four studies about lung ultrasound and chest computed tomography in post-COVID-19 patients.

Author	Year	Study Design	Patients	Age	Follow-Up Timing	Male	Patients with Lung Damage Resolution	Patients with Persistent Lung Damage	Standard Reference	LUS Scheme	Interpretation	Probe	Findings
Ramos Hernandez et al. [38]	2023	Observational and prospective multicentre study	233	62.4	3 ± 1 and 12 ± 1 months after hospital discharge	64.4%	65	168	CT/X-ray	14 areas	(0) = A lines; (1) = at least 3 vertical hyperechogenic artifacts; (2) = B lines tended toward coalescence; (3) = area of white lung or consolidation of the lung parenchyma. Altered LUS results were defined as all those with a lung score ≥ 1 while normal LUS findings were those with a score of 0 without the presence of artifacts in the pleural line.	2–5 MHz convex probe	The mean LUS score was 5.3 (SD = 5.1). LUS showed a sensitivity of 89.7%, specificity of 50%, and PPV of 90%. In the second visit, LUS showed a significant improvement compared to the first visit (5.8 ± SD 5.2 vs. 2.1 ± SD 3.8; *p* = 0.001). The sensitivity of LUS at this visit was 76%, specificity was 74%, PPV was 93%, and the AUC was 0.74.
Loke et al. [37]	2023	Prospective	21	52.1	Days 0 (D0), 41 (D41) and 83 (D83)	61.9%	-	-	CT	12 areas	(0) = A lines; (1) = vertical hyperechogenic artifacts; (2) = B lines tended toward coalescence; (3) = consolidation of the lung parenchyma.	Portable LUS probe	The mean LUS scores of patients on D41 and D83 were significantly lower, as compared to D0 of the study (2.9 ± 2.1 [D41] and 1.3 ± 1.3 [D83]) vs. 10.7 ± 3.3 [D0]; *p* < 0.001).
Russo et al. [39]	2022	Single centre, prospective observational study	74	65.0	6 months	73%	24	50	CT	12 areas	(0) = A-line pattern; <3 B lines can be present. (1) = at least 3 B lines in at least one scan of the region; the B lines are well separated and do not merge one in the other. Small subpleural consolidations ≤1 cm diameter and irregular pleural line. (2) = multiple, converging B-lines (white lung) in at least one scan of the region. Small subpleural consolidations ≤ 1 cm diameter and irregular pleural line. (3) = at least one consolidation with major vs. > 1 cm in at least one scan of the region.	Convex probe	Lung abnormalities were detected in 69.4%, with a median LUS score of 2 (IQR 0–5.25). When compared to LUS during hospitalization, a decrease in total score greater than 50% was observed in 76% of patients after 6 months. ROC showed an AUC of 0.85 (95% CI 0.76–0.93). LUS score < 2 can rule out fibrotic-like changes with a sensitivity of 0.92 (95% CI 0.73–0.99) and a specificity of 0.60 (95% CI 0.45–0.74).
Zimna et al. [40]	2024	Prospective observational study	72	58.0	3 months	62%	-	-	CT	12 areas	0 = Regular and continuous pleural line, A-lines; 1 = Irregular or broken pleural line, or consolidation ≤2.5 mm, or ≤3 B-lines; 2 = consolidation >2.5 mm ≤10 mm, or >3 B-lines; 3 = consolidation >10 mm, or pleural effusion, or coalescence B-lines, or “white lung” image.	Linear array transducer (3–13 MHz)	Fibrotic changes were observed in 41.6% of the patients. LUS score was significantly higher in patients with radiological evidence of fibrosis compared to those without (*p* = 0.000002, and *p* = 0.000000, in the two-follow-up timing, respectively). The mean ultrasound score in I e for the group exhibiting fibrotic features was 19.4 ± 5.7 points, whereas, for those without these features, it was 11 ± 6.6 points, and in II e, these scores were 16 ± 5.3 points and 6 ± 2.7 points, respectively. All patients with an ultrasound score below 9 points showed near complete regression of lung lesions on chest CT. The optimal LUS score for the detection of more than 10% of GGOs was 13 points, which combined the highest sensitivity of 0.964 and lowest false-positive rate of 0.262 (specificity 0.738; NPV 0.904; PPV 0.89; AUC: 0.94.

## Data Availability

The data can be requested from the corresponding author.

## Appendix A

Search strategies

**PubMed**

#1 “post COVID-19” [Supplementary Concept]
#2 “post Severe Acute Respiratory Syndrome Coronavirus 2” [Supplementary Concept]
#3 “post COVID-19” [Title/Abstract]
#4 “post SARS-COV-2” [Title/Abstract]
#5 “post novel coronavirus” [Title/Abstract]
#6 “post 2019-novel coronavirus” [Title/Abstract]
#7 “post coronavirus disease-19” [Title/Abstract]
#8 “post coronavirus disease 2019” [Title/Abstract]
#9 “post COVID19” [Title/Abstract]
#10 “post Novel CoV” [Title/Abstract]
#11 “post 2019-nCoV” [Title/Abstract]
#12 “post 2019-CoV” [Title/Abstract]
#13 OR/#1-12
#14 lung ultrasound* [Title/Abstract]
#15 lung POCUS [Title/Abstract]
#16 lung ultrasound [MeSH Terms]
#17 OR/#14-16
#18 “lung radiography, thoracic” [MeSH Terms]
#19 “lung computed tomography” [Title/Abstract]
#20 “lung radiograph*” [Title/Abstract]
#21 “lung imagin*” [Title/Abstract]
#22 OR/#18-#21
#23 #13 AND #17 AND #22

**Cochrane library**

#1 “post COVID-19”:ti,ab,kw
#2 “post SARS-COV-2”:ti,ab,kw
#3 “post Novel coronavirus”:ti,ab,kw
#4 “post 2019-novel coronavirus”:ti,ab,kw
#5 “post Novel CoV”:ti,ab,kw
#6 “post 2019-nCoV”:ti,ab,kw
#7 “post 2019-CoV”:ti,ab,kw
#8 “post coronavirus disease-19”:ti,ab,kw
#9 “post coronavirus disease 2019”:ti,ab,kw
#10 “post COVID19”:ti,ab,kw
#11 OR/#1-10
#12 MeSH descriptor: [Lung Ultrasonography] explode all trees
#13 (lung ultrasound*):ti,ab,kw
#14 (lung ultrasonography*):ti,ab,kw
#15 lung POCUS:ti,ab,kw
#16 OR/12-15
#17 #11 AND #16

**Embase**

#1. ‘post COVID-19’:ab,ti
#2. ‘post SARS-COV-2’:ab,ti
#3. ‘post novel coronavirus’:ab,ti
#4. ‘post 2019-novel coronavirus’:ab,ti
#5. ‘post coronavirus disease-19’:ab,ti
#6. ‘post coronavirus disease 2019’:ab,ti
#7. ‘post COVID19’:ab,ti
#8. ‘post novel cov’:ab,ti
#9. ‘post 2019-ncov’:ab,ti
#10. ‘post 2019-cov’:ab,ti
#11. ‘post coronavirus disease 2019’/exp
#12. OR/#1-11
#13. ‘lung ultrasound’/exp
#14. Lung pocus:ti,ab
#15. Lung ultrasound:ti,ab
#16. OR/#13-15
#17. #12 AND #16
